# Contribution of the ROS/IRE1α Signaling Pathway to Arsenic Trioxide-Induced Apoptosis and Autophagy-Associated Responses in BEAS-2B Cells

**DOI:** 10.3390/molecules31203787

**Published:** 2026-10-21

**Authors:** Jiaming Yuan, Yanhong Chen, Chenjuan Yao, Haiyan Wei, Yu Han, Gang Chen, Jianhua Qu

**Affiliations:** 1 Department of Occupational Medicine and Environmental Toxicology, School of Public Health, Nantong University, Nantong 226019, China; yuanjiaming120@126.com (J.Y.); chenchengzi1120@163.com (Y.C.); why1987@ntu.edu.cn (H.W.); hanyu@ntu.edu.cn (Y.H.); 2 Hangzhou Fuyang District Center for Disease Control and Prevention (Hangzhou Fuyang District Health Supervision Institute), Hangzhou 311400, China; 3 Heqing Community Health Service Center, Pudong New Area, Shanghai 201201, China; 4 Department of Oral Physiology, Tokushima University Graduate School of Biomedical Sciences, 3-18-15 Kuramoto, Tokushima 770-8504, Japan; ychenjuan@tokushima-u.ac.jp

**Keywords:** arsenic trioxide, IRE1α, ROS, apoptosis, autophagy-associated responses

## Abstract

Inorganic arsenic primarily originates from geological processes and human activities. Exposure to high concentrations of inorganic arsenic can cause diverse health problems, with lung injury being one of the main hazards. The present study sought to elucidate the cytotoxicity and underlying mechanisms of inorganic arsenic in BEAS-2B cells. The results showed that exposure of BEAS-2B cells to arsenic trioxide (As_2_O_3_) for 24 h resulted in a dose-dependent decline in viability. As_2_O_3_ triggered reactive oxygen species (ROS) production in BEAS-2B cells, and ROS contributed to increased p-IRE1α and p-eIF2α abundance relative to β-actin, along with GRP78 protein expression. The autophagy-associated and apoptotic responses induced by As_2_O_3_ in BEAS-2B cells were likewise associated with ROS. Further analysis revealed that increased p-IRE1α abundance relative to β-actin is involved in As_2_O_3_-triggered autophagy-associated responses and apoptosis in BEAS-2B cells. The antioxidant N-acetylcysteine (NAC) markedly attenuated As_2_O_3_-induced apoptosis and autophagy-associated responses and reduced p-IRE1α abundance relative to β-actin. Collectively, these findings indicate that the ROS/IRE1α pathway contributes to As_2_O_3_-triggered autophagy-associated responses and apoptosis in BEAS-2B cells.

## 1. Introduction

Inorganic arsenic, a well-known toxicant, exists broadly in the natural environment in various chemical forms. Inorganic arsenic can be detected in food crops as a result of absorption from water and soil, mainly existing as soluble arsenite (AsIII). Inorganic arsenic is also present in the atmosphere, primarily as particulate arsenic trioxide (As_2_O_3_) [1,2,3]. Currently, inorganic arsenic contamination has become a global public safety concern given its worldwide distribution. According to surveys, an estimated 94–220 million individuals globally suffer from chronic exposure to excessive arsenic via contaminated drinking water [4]. In Bangladesh, the groundwater arsenic concentration in the vast majority of districts exceeds 50 μg/L [5], and people are also exposed to arsenic through inhalation due to substantial arsenic accumulation in the air from coal combustion and industrial exhaust emissions. In certain regions, the annual average atmospheric arsenic concentration has been measured at up to 17.5 ng/m^3^, which is 2.9 times the limit established by the Ambient Air Quality Standards of both EU and China [6]. In the workplace, airborne arsenic concentrations are significantly higher, reaching milligram levels per cubic meter [7]. Thus, inhalation has become a major route of arsenic exposure both in the general population and occupational groups.

Inorganic arsenic exhibits multi-organ toxicity, with the lung being the primary target. Epidemiological evidence has established associations between arsenic exposure and respiratory disorders, including lung function impairment [8,9]. Animal experiments show that continuous arsenic exposure induces inflammatory activation and pulmonary fibrosis [10]. In mechanistic investigations, several potential explanations such as oxidative stress, DNA damage and cytotoxicity have been proposed for arsenic-mediated lung toxicity. Among these, oxidative stress injury is recognized as playing the most significant role [11,12,13].

Oxidative stress, manifested as supraphysiological accumulation of reactive oxygen species (ROS), can disturb the delicate redox balance essential for proper protein folding in the endoplasmic reticulum (ER), thereby leading to the ER stress. To cope with this stress, eukaryotic cells initiate a set of signaling cascades collectively referred to as the unfolded protein response (UPR). Three major branches of the UPR have been identified and are triggered separately by protein kinase RNA-like ER kinase (PERK), inositol-requiring enzyme 1α (IRE1α), and activating transcription factor 6 (ATF6) [14,15,16]. Among these three branches, PERK and IRE1α have attracted particular attention given their involvement in modulating autophagy and apoptosis. Once activated, PERK primarily targets eukaryotic translation initiation factor 2α (eIF2α) for phosphorylation. This subsequently upregulates certain gene transcripts associated with autophagy, apoptosis, antioxidant responses and amino acid metabolism [17,18]. IRE1α is a kinase and endoribonuclease. It promotes X-box binding protein 1 (XBP1) translation upon activation, and the newly synthesized XBP1 exerts its effects through transcriptional activation of diverse UPR target genes, resulting in enhanced ER protein-folding capacity as well as accelerated ER membrane biogenesis [19]. Activated IRE1α can also bind to tumor necrosis factor receptor-associated factor 2, leading to activation of the apoptosis signal-regulating kinase 1 (ASK1) pathway and its downstream target c-Jun N-terminal kinase (JNK). JNK activation is an important pro-apoptotic signal in response to IRE1α activation [20]. The UPR has a dual role in cell fate decision. It serves as an adaptive mechanism to protect cells from stress and restore homeostatic balance. Nevertheless, a severe or prolonged UPR can trigger apoptosis and autophagy [21].

Autophagy, a process characterized by clearing dysfunctional organelles and recycling metabolic components, is essential for maintaining cellular homeostasis. Excess autophagy, which is induced by various stress events including ER stress, has been implicated in lung disease [22]. Similarly, dysregulated apoptosis can lead to various pulmonary pathological conditions, including lung injury [23,24]. In mammalian cells, autophagy and apoptosis triggered by ER stress are associated with UPR signaling cascades, wherein oxidative stress is a critical determinant [25]. With respect to arsenic exposure, oxidative stress has been implicated as a key mediator of lung toxicity [26].

In our previous study, we found that As_2_O_3_ induces ROS generation [27] and triggers ER stress to activate the UPR in BEAS-2B cells [28]. However, the relationship between oxidative stress and UPR induced by As_2_O_3_ in BEAS-2B cells as well as the relationship between UPR and apoptosis/autophagy, remains unclear.

Therefore, the present work was undertaken to evaluate the impact of As_2_O_3_ on autophagy-associated responses and apoptosis in BEAS-2B cells, with a focus on oxidative stress, ER stress, and UPR signaling. In addition, we probed the participation of specific UPR sensors to further explore the molecular mechanisms behind As_2_O_3_-induced autophagy-associated responses and apoptosis.

## 2. Results

### 2.1. As_2_O_3_ Induces Apoptosis in BEAS-2B Cells

**Figure 1:**
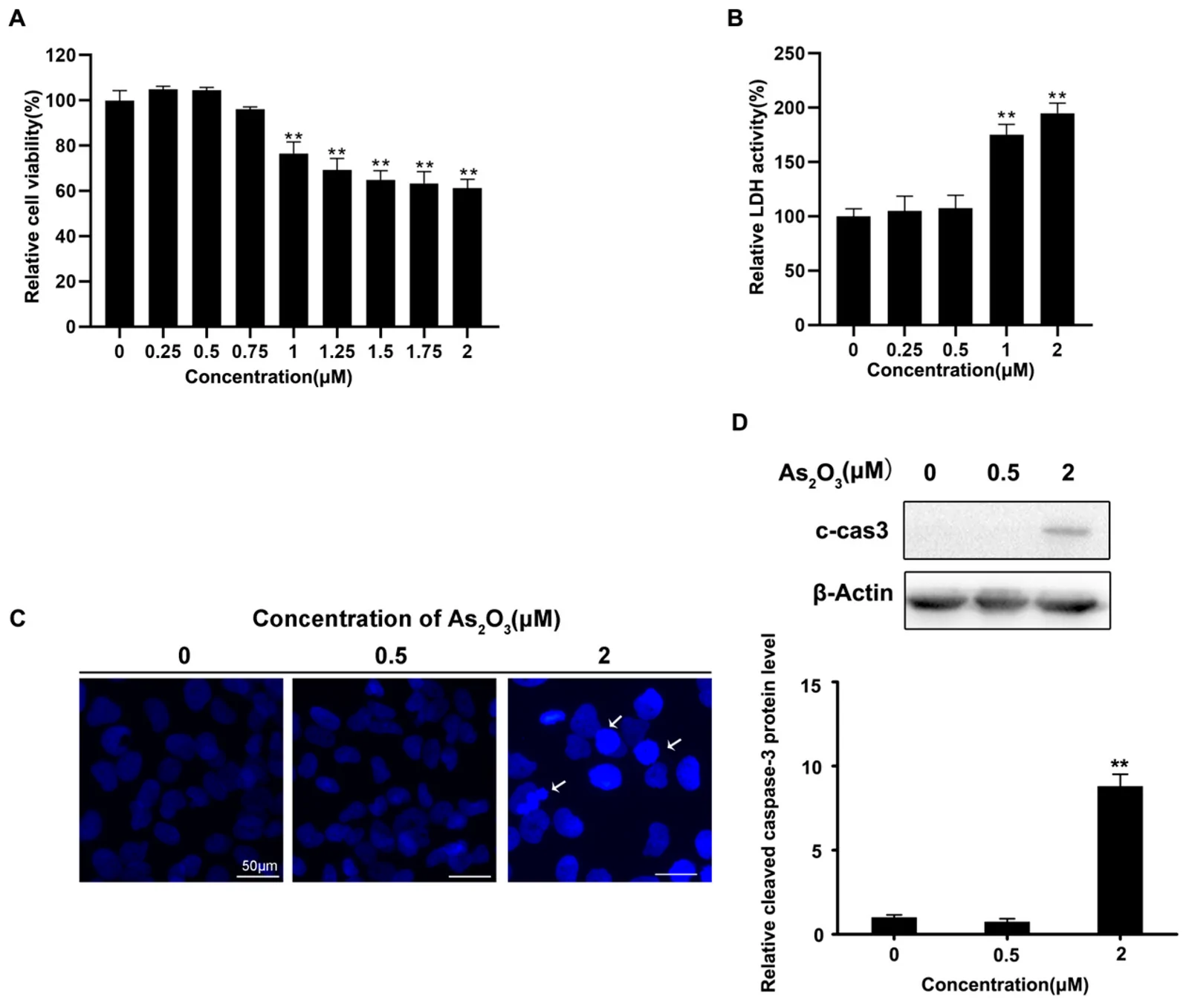
(**A**) Following 24 h treatment with increasing doses of As_2_O_3_ or PBS as a vehicle control, cell survival was quantified using CCK-8 assays. Six independent biological replicates for each concentration were prepared. (**B**) Following 24 h treatment with increasing doses of As_2_O_3_ or PBS as a vehicle control, BEAS-2B cell culture supernatants were collected for LDH activity assessment. Six independent biological replicates for each concentration were prepared. (**C**) BEAS-2B cells exposed to 0.5 or 2.0 μM As_2_O_3_ for a period lasting 24 h were stained with Hoechst 33342. Apoptotic nuclei, identified by shrinkage, condensation, and/or fragmentation, are indicated by arrows. Scale bar = 50 µm. (**D**) Following a 24 h challenge with As_2_O_3_ at 0.5 or 2.0 μM, BEAS-2B lysates were immunoblotted for cleaved caspase-3 and β-actin (loading control). Upper panel: Immunoblots for the indicated proteins. Lower panel: Quantitative densitometry of the blots shown in the upper panel, with signals normalized to β-actin. Data are mean ± SD, ** *p* < 0.01 vs. control.

To assess As_2_O_3_-triggered apoptosis in BEAS-2B cells, we initially employed the CCK-8 assay as a measure of cell viability. The cells were exposed to 0.25–2.0 μM As_2_O_3_ for 24 h. Here, 0.25–0.75 μM As_2_O_3_ did not alter BEAS-2B cell viability, whereas 1.0–2.0 μM As_2_O_3_ significantly reduced cell viability (Figure 1A). Lactate dehydrogenase (LDH) is a key enzyme released when cells die and can act as a marker of necrotic and late apoptotic cells. LDH release into the medium was quantified after the exposure of BEAS-2B cells to increasing As_2_O_3_ doses. The results showed that 1.0 and 2.0 μM As_2_O_3_ treatment in BEAS-2B cells resulted in a significant elevation in LDH levels (Figure 1B). Morphologically, apoptotic cells are characterized by cellular and nuclear shrinkage, chromatin compaction, and genomic DNA fragmentation. We assessed whether As_2_O_3_ induces apoptotic nuclear alterations in BEAS-2B cells using Hoechst 33342 staining. Cells maintained in normal medium or exposed to 0.5 µM As_2_O_3_ exhibited weak, homogeneous blue nuclear fluorescence, indicating a healthy nuclear state. In contrast, cells exposed to 2.0 μM As_2_O_3_ exhibited pronounced apoptotic nuclear features including condensed chromatin, shrunken nuclei, and brightly stained fragments (Figure 1C). Cleaved caspase-3 serves as a crucial “executioner” hallmark of the apoptotic process. Immunoblot analysis showed an upregulation of cleaved caspase-3 protein abundance in BEAS-2B cells following incubation with 2.0 μM As_2_O_3_ (Figure 1D). The above results suggest that apoptosis is involved in As_2_O_3_-mediated BEAS-2B cell death. The data presented in Figure 1 were newly generated for the present study and were not reused from our previous publications.

### 2.2. ROS Contribute to ER Stress and Apoptosis Induced by As_2_O_3_ in BEAS-2B Cells

**Figure 2:**
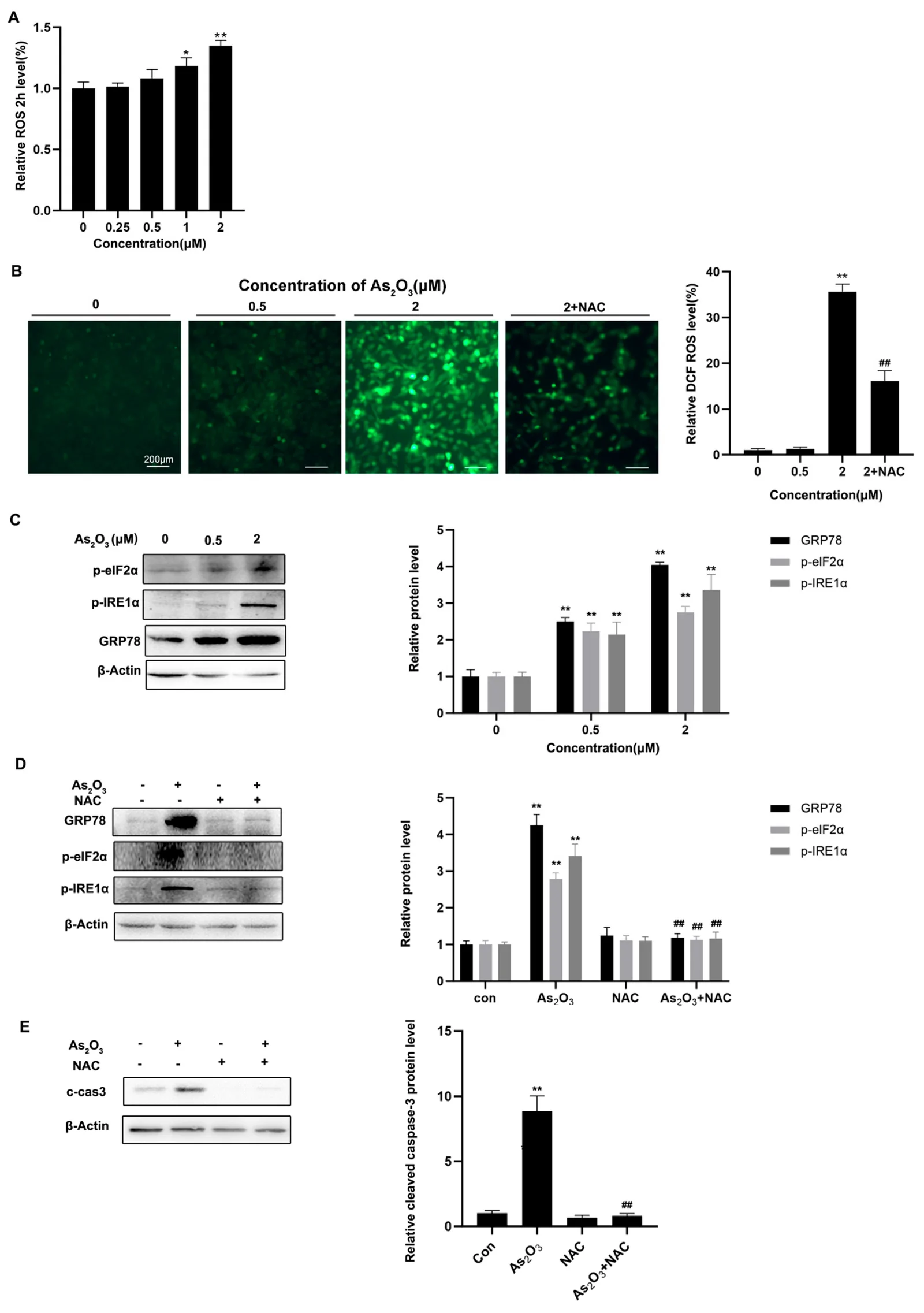
(**A**,**B**) Following exposure to increasing doses of As_2_O_3_ or PBS as a vehicle control for 2 h (**A**) or 24 h (**B**), ROS levels in BEAS-2B cells were assessed using a fluorometric microplate reader (**A**) and fluorescence microscopy (**B**) with DCFH-DA dye. In (**B**), a group treated with As_2_O_3_ plus NAC was also included. For (**B**), three independent biological replicates were performed. Three randomly selected fields per group were analyzed per experiment (at least 90 cells in total). Background was subtracted using a cell-free region of interest. Data were normalized to the vehicle control. Scale bar = 200 µm. Six independent biological replicates for each concentration were prepared (**A**). (**C**) Following a 24 h challenge with As_2_O_3_ at 0.5 or 2.0 μM, BEAS-2B lysates were immunoblotted for phospho-eIF2α, phospho-IRE1α, GRP78 and β-actin. Left panel: Immunoblots for the indicated proteins. Right panel: Quantitative densitometry of the blots shown in the left panel, with normalization of signals against β-actin (*n* = 3 independent biological replicates). (**D**,**E**) Following pretreatment with or without 5.0 mM NAC for 30 min followed by a 24 h challenge with 2.0 μM As_2_O_3_, immunoblot analysis of p-IRE1α, GRP78, p-eIF2α, cleaved-caspase 3, and β-actin was performed using BEAS-2B cell lysates. Left panel: Immunoblots for the indicated proteins. Right panel: Quantitative densitometry of the blots shown in the left panel, with normalization of signals against β-actin (*n* = 3 independent biological replicates). Data are mean ± SD, * *p* < 0.05 vs. control, ** *p* < 0.01 vs. control, ## *p* < 0.01 vs. As_2_O_3_ group.

Excessive ROS can trigger apoptosis through the mitochondria-mediated pathway by releasing cytochrome c and activating caspases. ROS is also involved in ER stress-associated apoptosis. To explore the mechanistic basis by which As_2_O_3_ induces apoptosis, we measured ROS levels in BEAS-2B cells after treatment with varying doses of As_2_O_3_ for 2 h. A marked elevation in ROS generation was detected with exposure to 1.0 μM and 2.0 μM As_2_O_3_ (Figure 2A). Intracellular ROS production in BEAS-2B cells was visualized using a fluorescence microscope after 24 h of As_2_O_3_ treatment. Enhanced intracellular green fluorescence was detected in cells in the 2.0 μM As_2_O_3_ group. However, negligible fluorescent signals were observed in the control and 0.5 μM As_2_O_3_ treatment group. In addition, NAC treatment markedly reduced the green fluorescence signal in cells exposed to 2.0 μM As_2_O_3_ (Figure 2B). ER stress triggers the UPR via transmembrane sensors located in the ER membrane. To investigate whether As_2_O_3_ triggers ER stress, the protein levels of GRP78 (an ER chaperone and a marker of UPR activation), as well as p-IRE1α and p-eIF2α abundance relative to β-actin, were assessed. Following 24 h treatment of BEAS-2B cells with 0.5 and 2.0 μM As_2_O_3_, p-IRE1α and p-eIF2α abundance relative to β-actin was significantly increased, and GRP78 protein levels also increased (Figure 2C). To assess ROS involvement in As_2_O_3_-triggered ER stress, BEAS-2B cultures were subjected to 30 min pre-treatment with 5.0 mM NAC prior to 24 h of As_2_O_3_ exposure at 2.0 μM. NAC markedly suppressed GRP78 expression, as well as p-IRE1α and p-eIF2α abundance relative to β-actin (Figure 2D). These observations indicate that ROS contributes to As_2_O_3_-elicited ER stress in BEAS-2B cells. Next, we explored involvement of ROS in As_2_O_3_-triggered BEAS-2B cell apoptosis. The results showed that NAC pretreatment reduced As_2_O_3_-induced cleaved caspase-3 expression (Figure 2E). These findings suggest that ROS plays an important role in BEAS-2B cell apoptosis resulting from As_2_O_3_ exposure. The data presented in Figure 2 were newly generated for the present study and were not reused from our previous publications.

### 2.3. ROS Contribute to As_2_O_3_-Induced Autophagy-Associated Responses in BEAS-2B Cells

**Figure 3:**
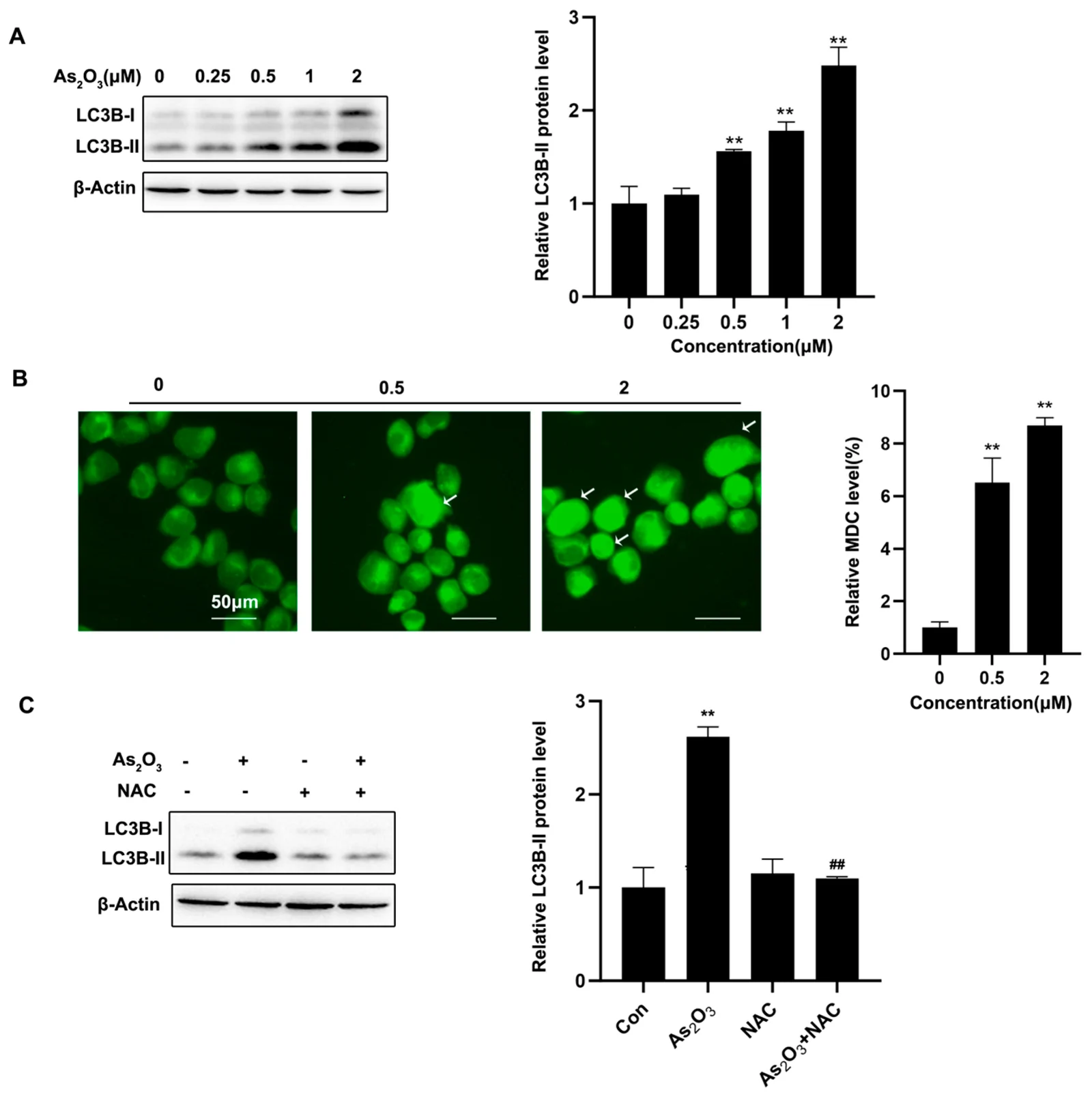
(**A**) Following a 24 h treatment with increasing doses of As_2_O_3_ or PBS as a vehicle control, BEAS-2B lysates were immunoblotted for LC3B and β-actin. Left panel: Immunoblots for the indicated proteins. Right panel: Quantitative densitometry of the blots shown in the left panel, with normalization of signals against β-actin (*n* = 3 independent biological replicates). (**B**) Following a 24 h challenge with As_2_O_3_ at 0.5 or 2.0 μM, autophagic vesicles in BEAS-2B cells were observed using MDC staining. Arrows indicate MDC-positive autophagic puncta. Three independent biological replicates were performed. Three randomly selected fields per group were analyzed per experiment (at least 90 cells in total). Background was subtracted using a cell-free region of interest. Data were normalized to the vehicle control. Scale bar = 50 µm. (**C**) Following pretreatment with or without 5.0 mM NAC for 30 min and a 24 h challenge with 2.0 μM As_2_O_3_, immunoblot analysis for LC3B and β-actin was performed using BEAS-2B cell lysates. Left panel: Immunoblots for the indicated proteins. Right panel: Quantitative densitometry of the blots shown in the left panel, with normalization of signals against β-actin (*n* = 3 independent biological replicates). Data are mean ± SD, ** *p* < 0.01 vs. control, ## *p* < 0.01 vs. As_2_O_3_ group.

Autophagy is generally cytoprotective, but harmful substances can induce lethal autophagy and lead to cell death. LC3B is a key protein in autophagy and LC3B-II levels are a key indicator of autophagosome formation. To investigate whether As_2_O_3_ induces autophagy-associated responses in BEAS-2B cells, the cultures were subjected to increasing doses of As_2_O_3_ for 24 h, and LC3B expression was evaluated with immunoblotting. Here, 0.5 μM treatment of BEAS-2B cells for 24 h led to an increased LC3B-II/β-actin ratio, with a further increase at 2.0 μM (Figure 3A). Monodansylcadaverine (MDC) accumulates as a fluorescent marker for the formation of autophagic vacuoles. Cells exposed to 0.5 μM and 2.0 μM As_2_O_3_ exhibited up-regulated MDC fluorescence intensity compared with control cells, with the most pronounced increase observed in the 2.0 μM group (Figure 3B). To assess whether ROS are involved in As_2_O_3_-triggered autophagy-associated responses in BEAS-2B cells, we exposed the cultures to 5.0 mM NAC for 30 min prior to 24 h exposure to 2.0 μM As_2_O_3_. NAC administration markedly reduced the LC3B-II/β-actin ratio triggered by As_2_O_3_ in BEAS-2B cells (Figure 3C). The reversal of this effect by NAC implicates ROS as potential upstream mediators of BEAS-2B cell autophagy-associated responses triggered by As_2_O_3_.

### 2.4. IRE1α Contributes to As_2_O_3_-Induced Apoptosis and Autophagy-Associated Responses in BEAS-2B Cells

**Figure 4:**
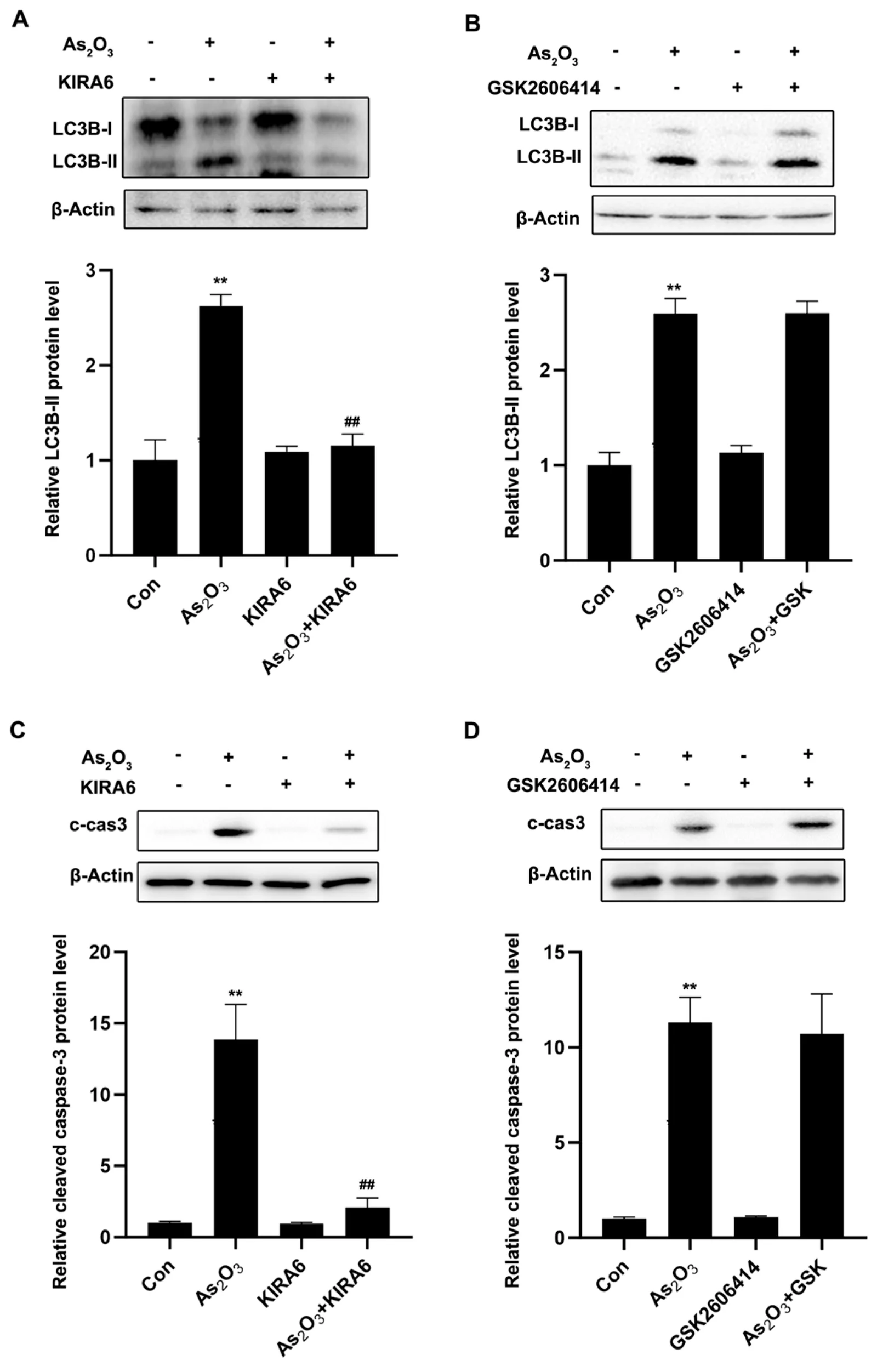
(**A**,**B**) Following 30 min of pretreatment with 1.25 μM KIRA6 or 0.1% DMSO (**A**) or 800 nM GSK2606414 or 0.1% DMSO (**B**) and 24 h challenge with 2.0 μM As_2_O_3_, immunoblot analysis of LC3B and β-actin was performed using BEAS-2B cell lysates. Upper panel: Immunoblots for the indicated proteins. Lower panel: Quantitative densitometry of the blots shown in the upper panel, with normalization of signals against β-actin (*n* = 3 independent biological replicates). (**C**,**D**) Following 30 min of pretreatment with 1.25 μM KIRA6 or 0.1% DMSO (**C**) or 800 nM GSK2606414 or 0.1% DMSO (**D**) and subsequent 24 h challenge with 2.0 μM As_2_O_3_, immunoblot analysis of cleaved caspase-3 and β-actin was performed using BEAS-2B cell lysates. Upper panel: Immunoblots for the indicated proteins. Lower panel: Quantitative densitometry of the blots shown in the upper panel, with normalization of signals against β-actin (*n* = 3 independent biological replicates). Data are mean ± SD, ** *p* < 0.01 vs. control (0.1% DMSO), ## *p* < 0.01 vs. As_2_O_3_ group.

These above observations support the notion that As_2_O_3_ may trigger ER stress, apoptosis, and autophagy-associated responses in BEAS-2B cells, with the involvement of ROS. KIRA6 serves as a potent IRE1α inhibitor, whereas GSK2606414 acts as a potent blocker of PERK. Both compounds are widely utilized to explore the pathological and physiological functions of the UPR in mammalian cells. To explore the functional connection between the UPR and the downstream autophagy-associated and apoptotic responses, BEAS-2B cells were maintained under three pretreatment conditions including 1.25 μM KIRA6, an equivalent volume of vehicle control, or 800 nM GSK2606414 for 30 min, followed by 2.0 μM As_2_O_3_ treatment for 24 h. KIRA6 markedly suppressed the As_2_O_3_-induced LC3B-II/β-actin ratio in BEAS-2B cells (Figure 4A). In contrast, GSK2606414 had no obvious effect on LC3B-II induced by As_2_O_3_ (Figure 4B). In similar results, KIRA6 effectively inhibited the level of cleaved caspase-3 induced by As_2_O_3_ (Figure 4C), whereas GSK2606414 did not significantly inhibit this effect (Figure 4D). These results suggest that increased p-IRE1α abundance relative to β-actin contributes to As_2_O_3_-induced autophagy-associated responses and apoptotic responses in BEAS-2B cells.

## 3. Discussion

**Figure 5:**
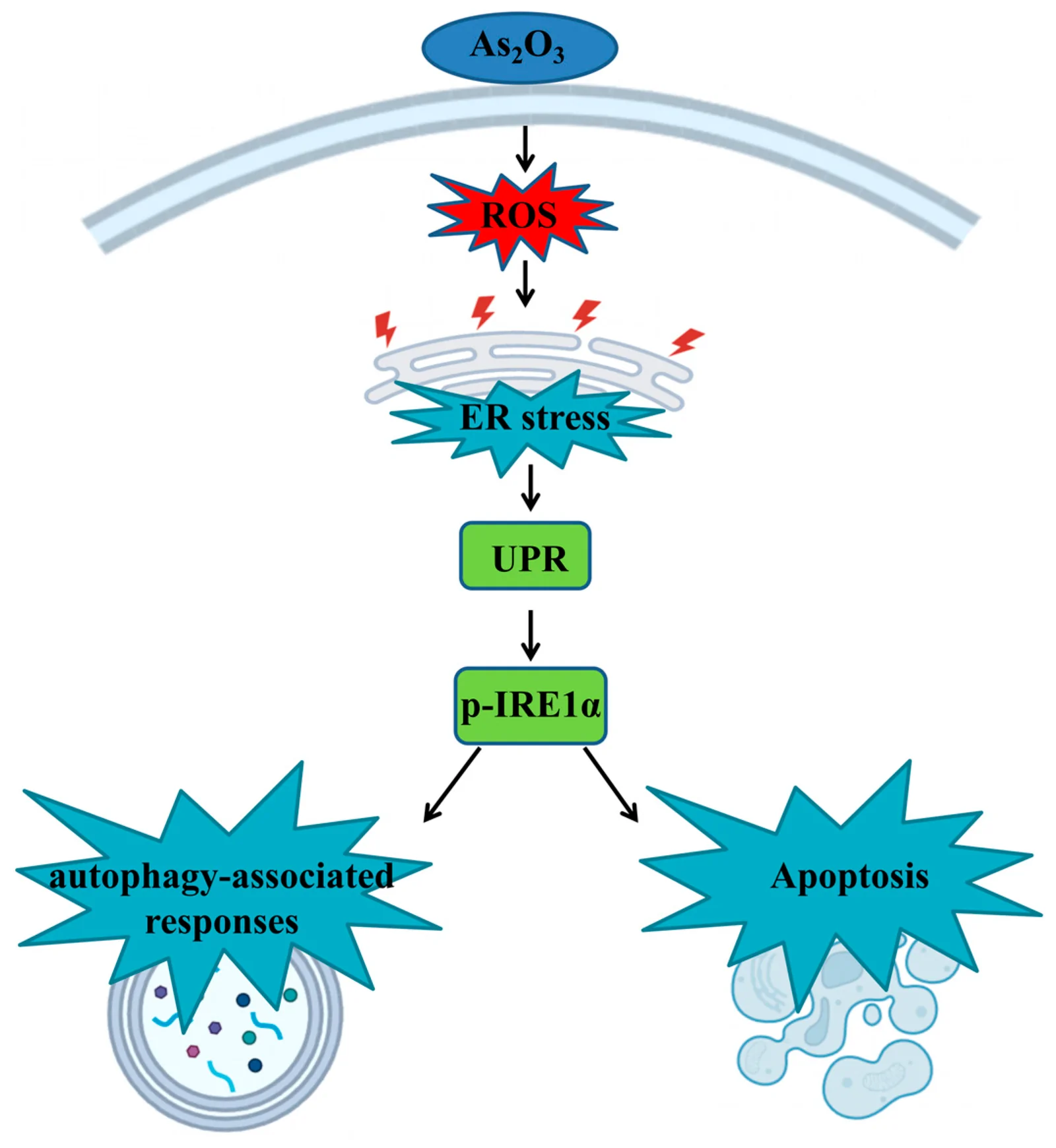
Proposed model of As_2_O_3_-induced ER stress and ROS/IRE1α signaling contributing to autophagy-associated responses and apoptosis in BEAS-2B cells. In this proposed model, As_2_O_3_ induces ROS production and activates ER stress, which subsequently stimulates the UPR. The IRE1α branch of the UPR contributes to autophagy-associated responses and apoptosis.

In recent years, an increasing number of researchers have been focusing on the relationship between inorganic arsenic toxicity and oxidative stress, but the underlying molecular mechanisms are yet to be explored [29,30]. In this research, we showed that As_2_O_3_ exposure evoked oxidative stress, ER stress, apoptosis, and autophagy-associated responses in BEAS-2B cells, with oxidative stress playing an important role in these processes. Moreover, we showed that IRE1α, a UPR component, contributed to autophagy-associated responses and apoptosis induced by As_2_O_3_ (Figure 5). These findings advance our understanding of As_2_O_3_ toxicity and provide mechanistic evidence underlying As_2_O_3_-induced lung injury.

Previous investigations have demonstrated that high doses of inorganic arsenic trigger intracellular ROS overproduction in mammalian cells. The mitochondrial electron transport chain serves as a primary route for this process, resulting in substantial ROS production. NADPH oxidase activation also participates in the generation of superoxide anions. Furthermore, arsenic metabolism itself represents an additional source of cellular ROS [31]. Consistent with these earlier reports, our data demonstrated that As_2_O_3_ at concentrations of 1.0 μM and 2.0 μM, but not at 0.5 μM and below, induced excessive ROS production in BEAS-2B cells. It should be noted that environmental arsenic exposure, particularly in endemic areas such as parts of Bangladesh where groundwater concentrations range from 0.05 to 2.5 mg/L [32], represents a significant public health concern. However, the present study is designed as an in vitro mechanistic investigation rather than a model of environmental exposure. The concentration of 2.0 μM As_2_O_3_ was selected to examine the molecular mechanism of As_2_O_3_ toxicity under controlled conditions.

Protein homeostasis is fundamental to cell survival. The production of misfolded or unfolded proteins under stressful conditions, as well as their continuous accumulation, results in ER stress, which subsequently triggers the UPR to restore protein folding homeostasis. ROS directly impair the free thiol groups that are critical for protein folding enzyme function, thereby promoting ER luminal accumulation of unfolded proteins, which subsequently trigger ER stress [33]. Additionally, ROS cause protein misfolding within the ER [34]. It has been widely reported that ROS overproduction stimulates the activation of ER stress [35,36]. Conversely, ROS inhibition attenuates ER stress and delays the activation of the UPR [37]. Supporting these earlier observations, our results show that As_2_O_3_ in BEAS-2B cells evokes oxidative stress and ER stress, whereas NAC, an antioxidant commonly used to probe ROS-dependent mechanisms, effectively suppresses ER stress as well as subsequent UPR activation. Nevertheless, the involvement of ROS was assessed primarily using DCFH-DA and NAC, and more specific oxidative stress measurements such as GSH/GSSG are needed in future studies to further confirm the upstream role of ROS.

Sustained ER stress triggers apoptosis primarily through UPR signaling. Among the UPR sensors, IRE1α is recognized as a key mediator of apoptosis in a wide range of cell types [38]. In our experiments, As_2_O_3_ treatment significantly increased p-IRE1α abundance relative to β-actin. IRE1α, when activated under excessive ER stress, recruits TRAF2 and ASK1, thereby triggering the JNK signaling cascade. The resulting phosphorylated JNK then promotes the activity of the pro-apoptotic Bcl-2 family. This drives their relocalization to mitochondria and subsequent cytochrome c efflux, which subsequently engages the caspase-9/3/7 cascade and leads to apoptosis [39,40,41]. In our previous work, 2.0 μM As_2_O_3_ increased JNK phosphorylation in BEAS-2B cells, and this effect was inhibited by the IRE1α inhibitors KIRA6 and APY29 [28]. In the current study, we further observed that KIRA6 markedly suppressed As_2_O_3_-induced apoptosis in BEAS-2B cells, as reflected in significantly reduced cleaved caspase-3 levels. NAC pretreatment also reduced p-IRE1α abundance relative to β-actin and inhibited apoptosis. In addition, we examined p-eIF2α abundance relative to β-actin to assess the involvement of the PERK branch, as eIF2α is a key downstream effector of PERK. The PERK/eIF2α/ATF4/CHOP cascade has been reported to trigger apoptosis under severe ER stress [42,43]. Herein, although p-eIF2α abundance relative to β-actin increased, GSK2606414 treatment did not affect As_2_O_3_-induced cleaved caspase-3 levels, indicating that PERK does not appear to be required under the experimental conditions examined. Taken together, these findings indicate that the ROS/IRE1α pathway contributes to As_2_O_3_-induced apoptosis in BEAS-2B cells and suggest that the downstream IRE1α/JNK pathway may also be involved, based on our previously published findings [28]. However, JNK signaling was not directly assessed in the present study.

Beyond apoptosis, ER stress-induced UPR also modulates autophagy, which is another major cellular stress response [44]. Autophagy functions as an intracellular recycling system that degrades damaged organelles and misfolded proteins to provide energy and building blocks for cell survival during stress or starvation. However, when the autophagic machinery is disrupted or overactivated, it can lead to cell death [45]. In this study, exposure of BEAS-2B cells to As_2_O_3_ significantly upregulated LC3B-II levels, suggesting autophagy-associated responses. This increase was attenuated by the IRE1α inhibitor KIRA6, suggesting that the IRE1α branch of the UPR contributes to As_2_O_3_-induced autophagy-associated responses. Such ER stress—autophagy crosstalk has been reported in yeast cells [46], *Caenorhabditis elegans* [47], and vertebrates [44]. Mechanistically, Ogata et al. reported that IRE1 deficiency or JNK inhibition suppressed autophagy, indicating the IRE1α/JNK pathway as an important mediator of ER stress-triggered autophagy [48]. Additional support for this observation comes from studies of an oxygen-induced retinopathy mouse model [49]. In contrast, PERK deficiency did not impair ER stress-induced autophagy, indicating that PERK is dispensable for this process [48]. Consistently, our results showed that a PERK pathway inhibitor did not affect As_2_O_3_-induced autophagy-associated responses. Thus, these findings suggest that the IRE1α branch of the UPR contributes to As_2_O_3_-triggered autophagy-associated responses in BEAS-2B cells, whereas PERK does not appear to be required under the conditions examined. However, our data only demonstrate an association between autophagy and apoptosis. Thus, whether autophagy promotes cell death or serves a protective role remains to be determined. Finally, autophagy was assessed primarily based on LC3B-II levels, which cannot alone distinguish between enhanced autophagosome formation and impaired autophagosomal degradation. Future studies are needed to confirm whether these changes reflect enhanced autophagic flux.

Our previous studies have shown that As_2_O_3_ induces ROS production and triggers ER stress to activate the UPR in BEAS-2B cells. The present study extends these findings by showing that As_2_O_3_ induces apoptosis and autophagy-associated responses in BEAS-2B cells and that the IRE1α branch of the UPR contributes to these responses. Taken together, these findings suggest that the ROS/IRE1α pathway contributes to As_2_O_3_-induced apoptosis and autophagy-associated responses in BEAS-2B cells, providing insight into the pulmonary toxicity of inorganic arsenic.

## 4. Materials and Methods

### 4.1. Cell Culture and Treatments

The BEAS-2B cell line was purchased from cell bank of the Chinese Academy of Sciences. Cryopreserved BEAS-2B cells were thawed and passaged for five generations. Cells at passages 6–12 were used in this study. BEAS-2B cells were maintained in the DMEM/F12 medium in the presence of 10% FBS (Gibco Life Technologies, New York, NY, USA), with the basal medium obtained from Corning Life Sciences (Manassas, VA, USA). All incubations were performed at 37 °C in a humidified chamber, and the gas mixture contained 95% air and 5% CO_2_. To investigate how As_2_O_3_ induces autophagy-associated responses and apoptosis in BEAS-2B cells, inhibitors including 1.25 μM KIRA6 (Cayman Chemical, Ann Arbor, MI, USA), 5.0 mM Nacetyl-cysteine (NAC, Sigma-Aldrich, St. Louis, MO, USA), and 800 nM GSK2606414 (Target Molecule, Boston, MA, USA), were employed. As_2_O_3_ (Merck and Co., Inc., Kenilworth, NJ, USA; purity > 99.95%) was dissolved in 1 N NaOH at 100 mM and further diluted to 5 mM in PBS as a stock solution, and the final treatment solution had a pH of 7.5. BEAS-2B cells were pretreated with these agents for 30 min, after which the cultures were subjected to a 24 h challenge with As_2_O_3_ at either 0.5 or 2.0 μM.

### 4.2. Cell Viability Assay

To determine the cytotoxic action of As_2_O_3_ against BEAS-2B cells, we used the CCK-8 method (Dojindo Laboratory, Kumamoto, Japan). In brief, BEAS-2B cells plated into 96-well plates were incubated overnight to reach approximately 90% confluence. The cultures were subsequently exposed to indicated doses of As_2_O_3_ for 24 h. Following the preceding procedure, 10 μL CCK-8 was introduced, and the cultures were left in a light-free environment for 2 h. A BioTek ELX800 microplate reader (BioTek Instruments, Winooski, VT, USA) was employed to read the optical density at 450 nm. The resulting data were converted to relative percentages, with untreated controls serving as the 100% reference.

### 4.3. Lactate Dehydrogenase (LDH) Leakage Assay

The release of LDH in BEAS-2B cells was detected using a commercial LDH assay kit (Nanjing Jiancheng Bioengineering Institute, Nanjing, China). In this experiment, we incubated the cells for 24 h with or without indicated doses of As_2_O_3_. The conditioned media were then harvested and subjected to a centrifugation step (100× *g*, 4 °C, 5 min). The supernatants were then transferred to 96-well plates for LDH content measurement on a microplate reader according to the kit’s instructions. The resulting data were normalized to untreated cells, whose LDH release was set at 100%.

### 4.4. Nuclear Staining with Hoechst 33342

BEAS-2B cells were plated onto glass coverslips either with or without the specified doses of As_2_O_3_ for 24 h. Following treatment, the cultures were rinsed with PBS to eliminate residual agents, and subsequently stained with Hoechst 33342 at a concentration of 5 µg/mL for 10 min. Staining was observed using a fluorescence microscope supplied by Leica Microsystems (Wetzlar, Germany). In cells undergoing apoptosis, the nuclei appeared as condensed or fragmented.

### 4.5. Western Blotting Analysis

BEAS-2B cells were harvested and disrupted on ice in RIPA buffer fortified with protease and phosphatase inhibitors (Roche Applied Sciences, Indianapolis, IN, USA; Beyotime Biotechnology, Shanghai, China) following the manufacturer’s protocols. Lysates were clarified using centrifugation (12,000 rpm, 15 min), and supernatants were harvested. Protein levels were then quantified using the standard BCA procedure (Beyotime Biotechnology, Shanghai, China). For immunoblotting, equivalent protein aliquots were mixed with SDS loading buffer, heated to 95 °C, and subjected to SDS-PAGE separation and the resolved proteins were then electrotransferred onto PVDF membranes using a wet transfer system (Immobilon-P, Merck Millipore, Darmstadt, Germany). Following a 2 h block of the membranes in non-fat milk at room temperature, overnight probing at 4 °C was then performed using specific primary antibodies against GRP78 (sc13539, Santa Cruz Biotechnology, Santa Cruz, CA, USA), cleaved caspase-3 (9664T, Cell Signaling Technology, Danvers, MA, USA), phospho-eIF2α (Ser51, 9721, Cell Signaling Technology, Danvers, MA, USA), phospho-IRE1α (Ser724, ab48187, Abcam, Cambridge, UK), LC3B (2775, Cell Signaling Technology, Danvers, MA, USA), and β-actin (A3854, Sigma-Aldrich, St. Louis, MO, USA), all at the recommended dilutions. Following extensive washes, blots were exposed to HRP-labeled secondary antibodies (7074 or 7076, Cell Signaling Technology, Danvers, MA, USA; or A0192, Beyotime Biotechnology, Shanghai, China). Immunoreactive bands were visualized using ECL detection kits (Millipore, Bedford, MA, USA) and signals were recorded on a Tanon 5500 imager (Tannon, Shanghai, China). Band intensities were quantified using Image J software (version 1.4.3).

### 4.6. Cellular ROS Determination

ROS generation was monitored using DCFH-DA fluorescence (Beyotime, Shanghai, China) following the specifications supplied by the manufacturer. For the quantitative assay, BEAS-2B cells (2 × 10^5^/well) were seeded and maintained on 96-well plates and left to attach for 12 h, followed by exposure to indicated concentrations of As_2_O_3_ for 2 h. Following gentle washing with PBS twice, the cells in each well were loaded with 200 μL of 10 μM DCFH-DA. Then, the plates were incubated at 37 °C for 20 min to allow sufficient dye uptake. Unbound probe was removed with two additional PBS rinses, and the cells were then equilibrated in 100 μL of culture medium for 10 min. Fluorescence intensities were subsequently quantified using an EnSpire 2300 Plate Reader (Perkin Elmer, Waltham, MA, USA) at 480 nm (excitation) and 530 nm (emission). For fluorescent imaging, BEAS-2B cells on coverslips were subjected to 0.5 μM and 2.0 μM As_2_O_3_ for 24 h. The cultures were then labeled using 200 μL 10 μM DCFH-DA in a CO_2_ incubator for 30 min. Image was observed using a Leica fluorescence microscope (Leica Microsystems, Germany). The fluorescence intensity was quantified using Image J software. Identical imaging settings were used for all groups. For each experiment, three randomly selected fields per group were analyzed, and at least 30 cells per condition were quantified (approximately 90 cells in total across three independent biological replicates). Background was subtracted using a cell-free region of interest. Data were normalized to the vehicle control.

### 4.7. Detection of Autophagy-Associated Responses Using Monodansylcadaverine (MDC) Staining

To visualize autophagic vacuoles, BEAS-2B cells were stained with MDC following As_2_O_3_ treatment. Briefly, cells on coverslips were exposed to 0.5 or 2.0 μM As_2_O_3_ for a period lasting 24 h, followed by a 10 min incubation with 0.05 mM MDC (Beyotime, Shanghai, China) at 37 °C. After three washes with PBS, stained cells were imaged using a confocal microscopy (Leica Microsystems, Germany). The fluorescence intensity was quantified using Image J software. Identical imaging settings were used for all groups. For each experiment, three randomly selected fields per group were analyzed, and at least 30 cells per condition were quantified (approximately 90 cells in total across three independent biological replicates). Background was subtracted using a cell-free region of interest. Data were normalized to the vehicle control.

### 4.8. Statistical Analysis

Values were shown as mean ± SD. Intergroup differences were evaluated using one-way ANOVA followed by Tukey’s post hoc test. *p* < 0.05 was considered significant. Independent biological replicates refer to experiments performed using independently cultured cells on separate experimental occasions.

## Data Availability

The original contributions presented in this study are included in the article. Further inquiries can be directed to the corresponding authors.

## Abbreviations

The following abbreviations are used in this manuscript:

As_2_O_3_	arsenic trioxide
NAC	N-acetyl cysteine
ROS	reactive oxygen species
ER	endoplasmic reticulum
UPR	unfolded protein response
PERK	protein kinase RNA-like ER kinase
IRE1α	inositol-requiring enzyme 1α
ATF6	activating transcription factor 6
eIF2α	eukaryotic translation initiation factor 2α
XBP1	X-box binding protein 1
ASK1	apoptosis signal-regulating kinase 1
JNK	c-Jun N-terminal kinase
LDH	lactate dehydrogenase
MDC	monodansylcadaverine
